# Case report: The diagnostic pitfall of Warthin-like mucoepidermoid carcinoma

**DOI:** 10.3389/fonc.2024.1391616

**Published:** 2024-06-20

**Authors:** Ying Yang, Zi Lei, Yixu Lang, Li Wu, Jun Hu, Shiyue Liu, Zaoxiu Hu, Guoqing Pan

**Affiliations:** ^1^ Department of Pathology, The First Affiliated Hospital of Kunming Medical University, Kunming, China; ^2^ Department of Pathology, The Chinese Medicine Hospital of Zhaotong, Zhaotong, China; ^3^ Department of Radiology, The First Affiliated Hospital of Kunming Medical University, Kunming, China; ^4^ Department of Pathology, The Third Affiliated Hospital of Kunming Medical University, Kunming, China

**Keywords:** Warthin-like mucoepidermoid carcinoma, mucoepidermoid carcinoma, salivary gland, Warthin Tumor, MAML2

## Abstract

Warthin-like mucoepidermoid carcinoma (WL-MEC) is a newly reported variant of mucoepidermoid carcinoma. Its histological feature is easy to confused with metaplastic Warthin Tumor, and its relationship with Warthin tumor in histogenesis is controversial. In this study, we presented two cases of WL-MEC, discussing their clinicopathological and molecular features. Notably, one case was initially misdiagnosed during the first onset of the tumor. Case 1 was a 60-year-old female with a mass in the right parotid gland. Case 2 featured a 29-year-old male who developed a lump at the original surgical site 6 months after a “Warthin tumor” resection from the submandibular gland. Histologically, both tumor exhibited a prominent lymphoid stroma and cystic pattern, accompanied by various amounts of epithelial nests composed of squamoid cells, intermediate cells and mucinous cells. The characteristic eosinophilic bilayer epithelium of Warthin tumor was not typically presented in either case. Both cases tested positive for *MAML2* gene rearrangement. To contextualize our findings, we conducted a comprehensive review of forty-eight WL-MEC cases documented in the English literature, aiming to synthesizing a reliable differential diagnostic approach. WL-MEC is a rare yet clinically relevant variant, posing a diagnostic pitfall for pathologists. Our study underscores the importance of a meticulous evaluation of both clinical and histological features, coupled with the detection of *MAML2* rearrangement, as a credible method for distinguishing WL-MEC from other benign and malignant lesions, particularly metaplastic Warthin tumor.

## Introduction

Mucoepidermoid carcinoma (MEC) is one of the most prevalent salivary gland malignancies, with a broad age distribution and a peak incidence in the second decade of life (1). Histologically, MEC manifests as a composition of squamoid cells, intermediate cells and mucinous cells, encompassing distinctive histological variants, such as the eosinophilic, clear cell and sclerosing variant. While MEC with classical morphology presents minimal diagnostic challenges, the identification of rare variants poses a complexity, often leading to confusion with other benign and malignant salivary gland lesions. Genetically, 55–82% of MECs harbor a *CRTC1/3-MAML2* gene fusion resulting from the translocation t(11;19) (q21;p13) (2, 3). This genetic aberration has emerged as a well-established and recognized molecular hallmark of MEC (4). The integration of *CRTC1/3-MAML2* gene fusion into the molecular profile of MEC enhances our understanding of its pathogenesis and provides a valuable diagnostic marker in the evaluation of salivary gland lesions.

Warthin tumor (WT) is the second most common salivary gland neoplasm, accounting for approximately 5–15% of all salivary gland tumors. Predominantly observed in elderly men with a history of smoking, WT almost always occurs in the parotid gland (1). Microscopically, it is characterized by a polycystic growth pattern, bilayered eosinophilic epithelium, and an abundant lymphoid stroma. Typical MEC is very different from typical WT in terms of microscopic morphology, and it is almost unnecessary to include WT in the differential diagnosis of MEC. However, diagnostic challenges arise when confronted with rare MEC variants and metaplastic WT.

In 2011, García et al. (5) reported 5 cases of MEC with Warthin-like stroma in a case series. Subsequently, in 2015, Ishibashi et al. coined the term “Warthin-like mucoepidermoid carcinoma”(WL-MEC) to designate this entity for the first time (6). Due to its intricate relationship with WT in histogenesis and morphological similarity to various benign and malignant tumors of the salivary gland, particularly metaplastic WT, this newly reported neoplasm has received extensive attention. Nevertheless, the diagnostic and differential diagnostic criteria for WL-MEC have not well developed owning to its rarity. Herein, we report 2 cases of WL-MECs and conduct a comprehensive review of 48 cases documented in the literature. The objective is to summarize the clinicopathological features and elucidate diagnostic considerations, thereby enhancing our comprehension of this uncommon variant and mitigating the risk of misdiagnosis.

## Case presentation

### Case 1

A 60-year-old female with no history of smoking was admitted to the hospital because of a mass under the right ear for 4 days. Physical examination revealed a 3×2.5 cm solid mass in the parotid gland region, characterized by its firm texture, mobility, well-defined borders, and absence of tenderness. Skin temperature over the mass was within the normal range.

**Figure 1:**
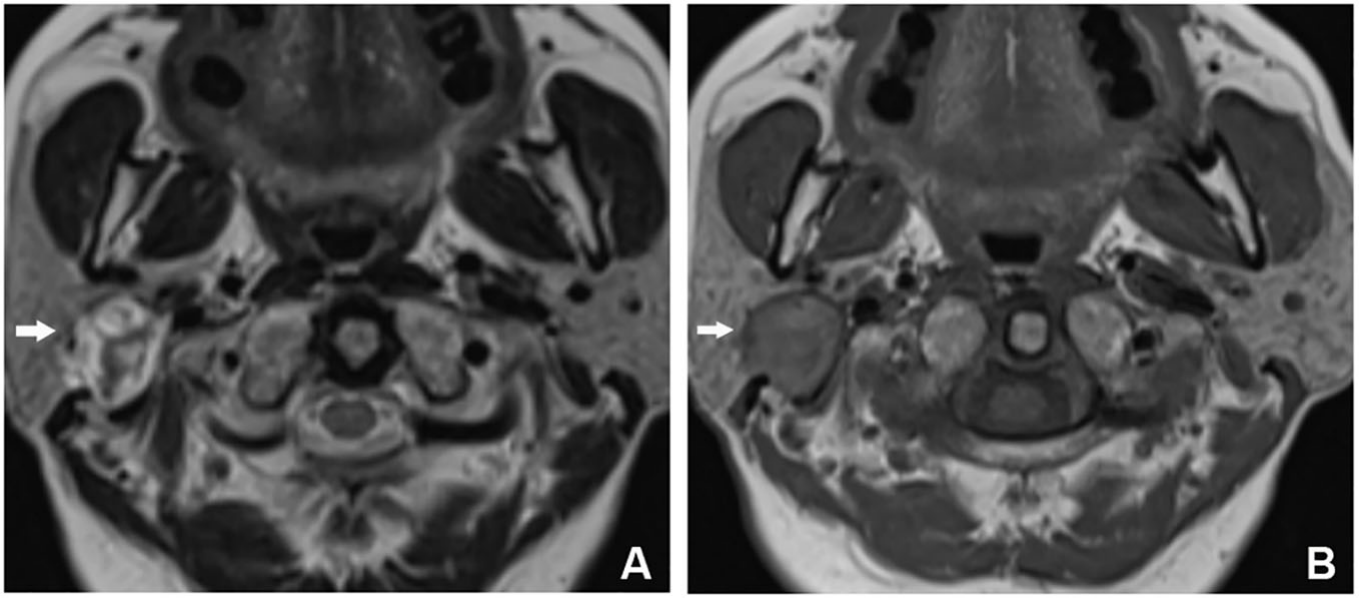
MRI findings for Case 1. **(A)** T2WI showed that the lesion was located in the deep lobe of the parotid gland, with a round shape and mixed long T2WI signals. There were multiple short T2WI signals on the interior, and short T2WI signal rings, which were locally discontinuous, were seen at the edge of the lesion. **(B)** The lesion was iso-/slightly short on T1WI, with a long T1WI signal ring, which was locally discontinuous, at the edge of the lesion.

Ultrasound examination disclosed a solid mass in the deep lobe of the right parotid gland, displaying uneven internal echoes. Preliminary considerations leaned towards pleomorphic adenoma. Magnetic resonance imaging (MRI) depicted a mass in the deep lobe of the right parotid gland, exhibiting mixed long/short T2 and iso-/slightly long T1 signals. Diffusion-weighted imaging (DWI) showed mixed signals, and enhanced scanning revealed conspicuous uneven enhancement. Septation enhancement was noted within the mass, and localized incomplete capsule formation was observed (**Figure 1**). The patient then received excision of lesions of parotid gland and parapharyngeal space + parotidectomy III–IV + facial nerve dissection + myocutaneous flap transfer.

**Figure 2:**
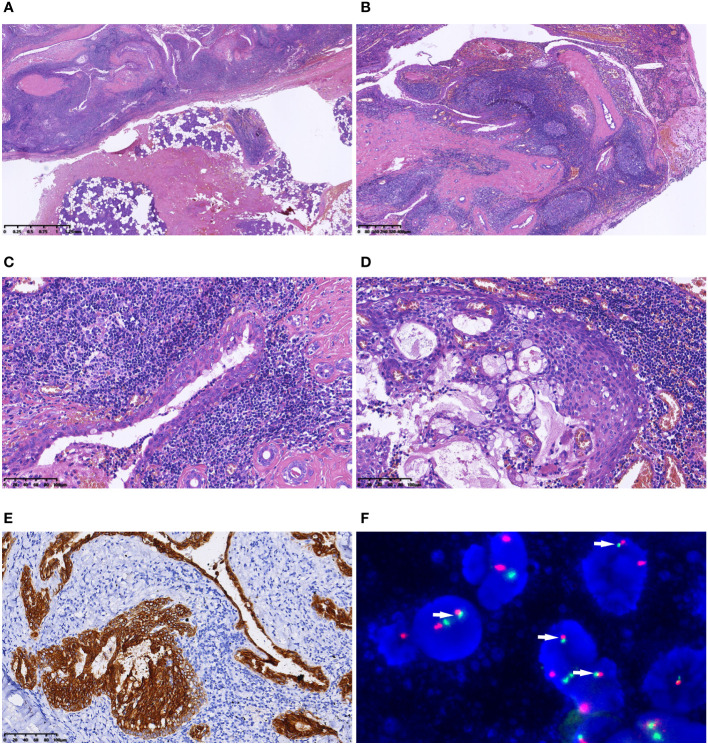
Microscopic features of Case 1. **(A)** The tumour was well defined; **(B)** Under a low-magnification microscope, the tumour exhibited polycystic structure. The cyst wall was lined with stratified epithelium, and the subepithelial stroma demonstrated dense lymphocytic arrangement, forming lymphoid follicles; **(C)** Epithelial cells, polygonal in shape with eosinophilic cytoplasm, exhibited a crowded pattern devoid of obvious polarity. Scattered mucus cells were interspersed in the epithelium; **(D)** Nested epithelial hyperplasia was evident in some areas, consisting of squamoid cells, mucous cells and intermediate cells; **(E)** Immunohistochemistry demonstrated positive expression of CK7 in the epithelial components; **(F)** FISH indicated the fusion signals of the *MAML2/CRTC1* gene (arrow).

Grossly, the surgical resection specimen comprised irregular grey−red tissue measuring 3.5×2×1 cm, displaying a combination of solid and cystic appearance on the cut surface. Histologically, the tumor exhibited well-defined and complex cystic architecture (**Figure 2A**). The cyst wall was lined with stratified epithelium, and the subepithelial stroma demonstrated densely arrangement of lymphocytes with formation of lymphoid follicles (**Figure 2B**). Epithelial cells, polygonal in shape with eosinophilic cytoplasm, exhibited a crowded pattern devoid of obvious polarity. The nuclei were round or oval with small nucleoli. Scattered mucus cells were interspersed in the epithelium (**Figure 2C**). In some areas, sheet-like epithelium hyperplasia was observed, comprising squamoid cells, mucous cells, and intermediate cells (**Figure 2D**). Mitotic figures were infrequent, with an absence of necrosis, nerve, and vascular invasion. Immunohistochemistry demonstrated positive expression of CK7, CK8, and CK18 in the epithelial components (**Figure 2E**). Squamoid cells and intermediate cells expressed CK5/6, P63 and P40, while β-catenin localized to the cell membrane. The Ki-67 index was approximately 1–2%, and calponin expression was absent. Fluorescence *in situ* hybridization (FISH) revealed *MAML2/CRTC1* gene fusion (**Figure 2F**). No metastasis was found in the resected four lateral neck level II lymph nodes. Subsequent to a 7-month follow-up, no recurrence or metastasis was observed.

### Case 2

A 29-year-old non-smoking male sought admission to the hospital because of “tumor recurrence 6 months after submandibular gland tumor resection”. Six months prior to admission, he had undergone submandibular gland tumor resection at another medical facility, with the postoperative pathological diagnosis indicating WT. On physical examination, a 3 cm-long surgical scar was seen on the left mandible, accompanied by a non-tender, non-ulcerated 3×2 cm mass.

Ultrasound examination revealed a 2.7×1.0 cm mass in the left submandibular gland, displaying an oval shape, clear boundaries, hypoechoic interior, uneven distribution, slightly enhanced posterior echo, and abundant blood flow signals. MRI scan illustrated a mass in the left submandibular region, characterized by iso/high signal intensity on T1WI and slightly high signal intensity on T2WI, with uneven enhancement. The lesion exhibited close proximity to the anterior border of the left submandibular gland, prompting consideration of malignancy. Excision of submandibular gland and tumor and functional neck dissection were performed.

**Figure 3:**
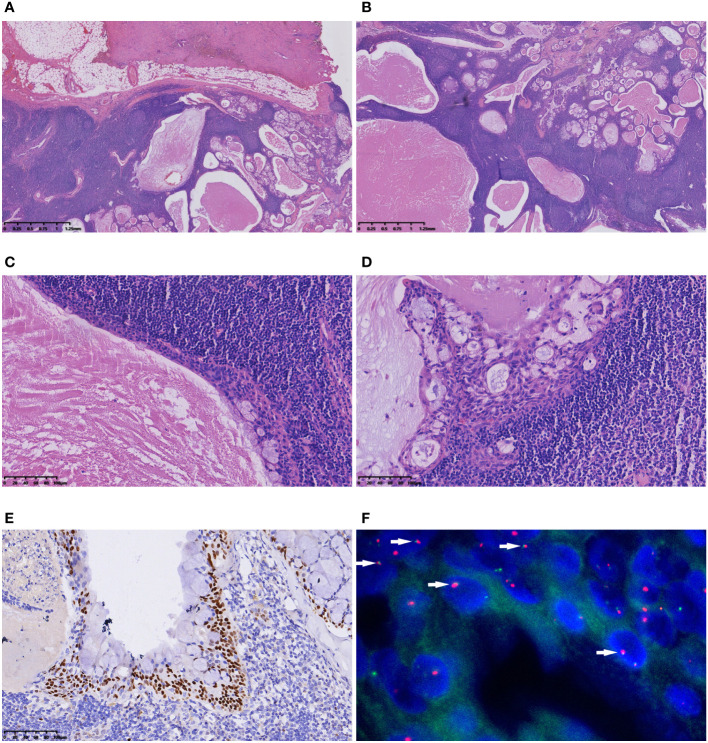
Microscopic features of Case 2. **(A)** The tumour had a clear boundary; **(B)** The polycystic appearance under a low-magnification microscope. The lesion presented an epithelial lining and multiple cysts within a backdrop of dense lymphoid stroma; **(C)** The stratified, nonpolar epithelium interspersed with mucus-secreting cells was evident; **(D)** Proliferation of epithelial cells in some regions formed nests, comprising squamoid, intermediate, and mucinous cells; **(E)** P63 immunohistochemical staining highlighted squamoid cells and intermediate cells; **(F)** FISH showed the fusion signals of the *MAML2/CRTC1* gene (arrow).

Macroscopic examination unveiled a well-defined grey−yellow nodule with a 2 cm diameter, exhibiting clear demarcation (**Figure 3A**). Microscopically, the lesion shared similarities with the previously described Case 1, presenting an epithelial lining and multiple cysts within a background of dense lymphoid stroma (**Figure 3B**). The epithelium was stratified, nonpolar, and interspersed with mucus-secreting cells (**Figure 3C**). Proliferation of epithelial cells in some regions formed nests, comprising squamoid, intermediate, and mucinous cells (**Figure 3D**). Immunohistochemically, epithelial components expressed CK7, while squamoid cells and intermediate cells expressed P63, P40, and CK5/6 (**Figure 3E**). The Ki-67 index was 1%, and calponin and SMA were not expressed. FISH revealed fusion of *MAML2/CRTC1* (**Figure 3F**). A lymph node was present in the specimen, and no metastasis was identified. Retrospective examination of slides from the initial operation displayed nearly identical morphological features to those of the recurrent tumor, substantiating the diagnosis of WL-MEC. A subsequent 4-month follow-up post-reoperation revealed no signs of recurrence or metastasis.

## Discussion

In our comprehensive review of 48 cases of WL-MEC (including our cases), clinicopathological information was available for 43 cases (**Table 1**) (5–20). The age of onset for WL-MEC ranged from 10 to 75 years, with a median age of 47 years, presenting a wider age distribution and a younger median age compared to patients with WT. Of the reviewed cases, 15 were male and 28 were female, resulting in a male-to-female ratio of 1:1.87. Among the cases with available smoking history data, only 1 out of 10 patients had a history of smoking. In stark contrast to the exclusively parotid gland location of WT, 41 cases (95.4%) of WL-MEC occurred in the parotid gland, with one case (2.3%) each observed in the palate and submandibular gland. Four of 32 cases (12.5%) reported the presence of pain, while the remaining cases manifested as painless masses. Tumour size ranged from 1.0 to 6.5 cm, with an average of 2.6 cm. The majority of tumors exhibited a polycystic or cystic-solid mass appearance. Most of the tumors had clear boundary while 6/31 cases (19.4%) displayed poorly demarcated or focal invasion. Microscopically, polycystic epithelial hyperplasia with prominent lymphoid stroma was a recurrent observation. Cysts were lined by an eosinophilic bilayer or multilayer epithelium with mild cellular atypia. The cytoplasm was eosinophilic, with interspersed mucous-secreting cells. Epithelial nests, composed of squamoid, intermediate, and mucinous cells, varied in quantity within the tumors. Mitotic figures, necrosis, and neurovascular invasion were infrequent. Importantly, the characteristic eosinophilic bilayer epithelium of WT was absent in 25/34 cases (73.5%), and when locally present in the remaining 9 cases (26.5%), it was notably less tall, less oncocytic, and less polarized. Immunohistochemistry revealed that the epithelial components expressed glandular epithelial markers (such as CK7, CK8, and CK18) and squamous epithelial and basal cell markers (such as CK5/6 and P63). The Ki67 index was low. Except for 4 cases without genetic testing data, the remaining 44 cases (91.7%) were all positive for *MAML2* gene rearrangement.

**Table 1:** Clinicopathological features of Warthin-like mucoepidermoid carcinoma reported in the literature.

Author	Cases,n	Age	Sex	Location	Size (cm)	Smoking history	Symptoms	Border	Presence of classic bilayered epithelia	Gene fusion	Follow-up (months)
Noda et al. (7)	1	16	F	L,PG	NA	NA	PFM	CD	NO	Y	6 NED
Bieńkowski et al. (8)	2	30	F	R,PG	1	No	PLM	ID	NO	Y	NA
		51	F	R,PG	2	No	PFM	ID	NO	Y	NA
Bishop et al. (9)	6	42	M	PG	3.1	NA	NA	CD	NO	Y	7 NED
		33	F	PG	3.2	NA	NA	CD	NO	Y	20 NED
		53	F	PG	3.3	NA	NA	CD	NO	Y	NA
		51	M	PG	NA	NA	NA	CD	NO	Y	NA
		51	F	PG	1.2	NA	NA	CD	NO	Y	NA
		53	F	PG	2.5	NA	NA	CD	NO	Y	NA
Zhang et al. (10)	9	10	M	PG	2.3	NA	PLM	CD	NA	Y	NA
		20	M	PG	2.0	NA	PLM	FI	NA	Y	NA
		48	M	PG	2.5	NA	PLM	CD	NA	Y	98 NED
		75	F	PG	4.5	NA	PLM	CD	NA	Y	86 NED
		59	F	PG	2.0	NA	PLM	FI	NA	Y	74 NED
		33	M	PG	1.3	NA	PLM	CD	NA	Y	58 NED
		46	F	PG	1.7	NA	PLM	CD	NA	Y	32 NED
		70	F	PG	4	NA	PLM	CD	NA	Y	96 NED
		72	F	PG	3	NA	PLM	CD	NA	Y	166 NED
Zhang et al. (11)	1	56	M	L,PG	6.5	NA	Mass	NA	NO	Y	16 NED
Ishibashi et al. (6)	5	28	F	PG	2.0	NA	NA	NA	NO	Y	120 NED
		28	F	PG	2.5	NA	NA	NA	NO	Y	36 NED
		33	F	PG	1.4	NA	NA	NA	NO	Y	96 NED
		46	F	PG	4.0	NA	NA	NA	NO	Y	120 NED
		60	F	PG	4.0	NA	NA	NA	NO	Y	12 NED
Heatley et al. (12)	1	17	F	PG	NA	NA	mass	NA	focal	NA	RA 48
Balasubiramaniyan et al. (13)	1	56	F	L,PG	1.8	NA	PLM	FI	Y	NA	8 NED
Hegde et al. (14)	1	17	M	Palate	NA	NA	PFM	NA	Y	NA	1 NED
Daoud et al. (15)	2	13	F	L,PG	3.5	No	PLM	CD	No	Y	22 NED
		14	M	R,PG	4.1	No	Mass	FI	No	Y	RA 12 *
García et al. (5)	4	68	F	R,PG	3.0	NA	Mass	NA	Focal	Y	26 NED
		50	M	PG	2.9	NA	Mass	NA		Y	NA
		46	F	PG	1.5	NA	Mass	NA		Y	NA
		64	F	PG	2	NA	Mass	NA		Y	NA
Zhang et al. (16)	1	36	M	L,PG	1.6	No	PLM	CD	atypical	Y	12 NED
Basak et al. (17)	3	16	F	R,PG	3.5	NA	PLM	NA	Y	Y	28 NED
		27	M	L,PG	2.2	NA	Mass	CD	No	NA	27 NED
		53	M	R,PG	1.9	NA	Mass	CD	Y	Y	32NED
Lei et al. (18)	2	47	M	R,PG	2.2	No	PFM	CD	No	Y	31 NED
		67	F	L,PG	3.0	No	PLM	CD	No	Y	27 NED
Hang et al. (19)	2	53	F	R,PG	2.3	NA	PLM	CD	No	Y	NA
		53	F	L,PG	2.5	Y	PLM	CD	No	Y	NA
Nakano et al. (20)	5	NA	NA	NA	NA	NA	NA	NA	NA	Y	NA
Our study	2	60	F	R,PG	2.6	No	PLM	CD	No	Y	7 NED
		29	M	L,SMG	2.0	No	PLM	CD	No	Y	RA 6 *

CD, clear demarcation; F, female; FI, Focal infiltration; ID, indistinct demarcation; L, left; M, male; NA, not available; NED, no evidence of disease; PG, parotid gland; PLM, painless mass; PFM, painful mass; R, right; RA, recurrence after;SMG, submandibular gland.

*Recurrence pattern was normal MEC

*This is a recurrent case. There was no recurrence or metastasis during the 4-month follow-up after reoperation.

The differential diagnosis of WL-MEC includes a series of benign and malignant tumors and lesions.

### WT with metaplasia

WT is frequently observed in elderly male smokers and almost always occurs in the parotid gland. Metaplasia changes in WT are usually focal and accompanied by hemorrhage and fibrosis associated with previous operations, such as biopsy. The epithelium in WL-MEC, as shown in our review, is often multilayered, with crowded cells and variable degree of atypia, lacking the typical bilayer epithelial structure observed in WT which exhibits impressive tall columnar cell morphology, eosinophilic cytoplasm, and polar arrangement. Ishibashi et al. (6) and Zhang et al. (11) both considered the presence of classic bilayer eosinophilic epithelium as the morphological key to differentiating WT from WL-MEC. More importantly, *MAML2* gene rearrangement serves as a useful distinguishing factor. As for treatment, WT has no risk of recurrence and metastasis, and only requires local resection.

### Lymphoepithelial carcinoma

Lymphoepithelial carcinoma is an undifferentiated cancer, characterized by epithelial nests and sheets distributed in lymphoid stroma, lacking polycystic structures observed in WL-MEC. The epithelium exhibits evident atypia, with vesicular nuclei, prominent nucleoli and abundant mitotic figures. Additionally, the occurrence of this carcinoma is related to Epstein-Barr (EB) virus infection.

### MEC occurs in WT (MEC ex WT)

Seifert et al. (21) referred to this as the malignant transformation of WT, proposing the following diagnostic criteria: i. pre-existence of a benign WT; ii. existence of transitional zones from benign oncocytic to malignant epithelium; iii. the infiltrating growth in surrounding lymphoid tissue; and iv. exclusion of metastasis.

### Benign lymphoepithelial lesions

Also known as lymphoepithelial sialadenitis, it occurs due to diffuse lymphocyte infiltration caused by atrophy of salivary gland tissue. Proliferation of the ductal epithelium and myoepithelium forms characteristic epithelial myoepithelial islands, lacking cellular atypia. The occurrence of these lesions is associated with Sjögren’s syndrome (22).

In addition, WL-MEC also needs to be differentiated from lymphoepithelial cysts, sebaceous lymphadenoma, squamous cell carcinoma with lymphocytic infiltration, and cystic lymph node metastases such as metastatic Warthin tumor-like thyroid carcinoma and metastatic squamous cell carcinoma.

The relationship between MECs and WTs has been a matter of debate for a long time. Squamous metaplasia and mucoid metaplasia are not uncommon morphological changes in WT, and the proportion of metaplasia reported in the literature ranges from 0.2% to 22% (23). When this metaplasia is extensive, it tends to be very similar to MEC in morphology. The occurrence of squamous and mucoid metaplasia in WT, coupled with reports of MECs arising in WT (MEC ex WT) (24, 25), has fueled speculation regarding their interconnection. Previous studies found that *MAML2* gene rearrangement occurs in a small number of WT cases, and some scholars have speculated that WT is a precursor of MEC^8^. Rotellini et al. (26) also detected *MAML2* gene rearrangement in 2 of 8 cases of WT with metaplasia. Accordingly, they assumed that the metaplastic changes in WT may be a sign of transformation of WT to MEC. However, recent studies have failed to detect *MAML2* gene rearrangement in larger samples of patients with WT (8). In addition, using FISH, Ishibashi et al. did not find *MAML2* heterogeneity in different morphological regions of whole tumor tissue sections of metaplastic WTs (6). The nomenclature for WL-MEC clarifies this confusion, and the current view is that metaplastic WT with *MAML2* gene rearrangement should be reclassified as WL-MEC (10). Therefore, the presence of *MAML2* gene rearrangement becomes the key to distinguishing WL-MEC from WT with metaplasia (8). This perspective challenges the recognition of MEC ex WT. Nevertheless, given the limited number of case reports, it is not clear whether the distinction between MEC ex WT and WL-MEC has any clinical significance. However, when squamous and myxoid metaplasia occurs in WT, vigilance should be exercised, and *MAML2* gene testing is advisable to exclude the possibility of malignancy (17).

Currently used histological grading systems for MEC include the Armed Forces Institute of Pathology system, modified Healey grading systems, the Brandwein system, and the Memorial Sloan Kettering system (20). Low- and medium-grade MECs exhibit less aggression and a better prognosis, typically treated with total parotidectomy and, if necessary, neck dissection. High-grade MECs may necessitate adjuvant radiotherapy (1, 8). A study by Behboudi et al. showed that MECs with *MECT1–MAML2* gene fusion had a better prognosis (27). Notably, WL-MEC, characterized as a low-grade MEC regardless of which grading system is used, exhibits a favorable prognosis. Among the 30 patients with follow-up records that we reviewed, only 3 (10.0%) developed recurrence after 6, 12 and 48 months during a median follow-up of 28 months (1–166 months). No case died of the disease. Therefore, as a low-grade malignant tumor, total parotidectomy with or without neck dissection was recommended for WL-MEC. Highly invasive treatments should be approached with caution.

Since malignant salivary tumors represent a diagnostic challenge because of their rarity and morphologic overlap, machine learning techniques have been applied to the field of pathology to improve diagnostic performance in recent years. Researches revealed promising results, although most applications are in developmental phase. However, in one research, mucoepidermoid carcinomas fell in one of the three histological types that easy to be misclassified by the tree-based machine learning model (28). For this special malignant tumor, more research is still needed.

The *CRTC1/3-MAML2* fusion in MEC has been well recognized for many years (2). The fusion encodes a chimeric protein in which the Notch-binding domain of MAML2 is replaced by the CREB-binding coiled-coil domain of CRTC1, and activates transcription of the Notch target gene HES1 independently of Notch ligand (3). Behboudi et al. demonstrated that the *CRTC1-MAML2* fusion is a useful marker in predicting the biological behavior of MECs (27). More recently, the study of Chen and colleagues provided direct evidence for *CRTC1-MAML2* as a key driver for MEC development and validated *CRTC1-MAML2* as a therapeutic target for patients with MEC (29). These studies provide a new direction for the accurate prognosis and treatment of MEC, including WL-MEC.

## Conclusion

In conclusion, WL-MEC stands as a rare and clinically relevant variant, posing a diagnostic challenge for pathologists. The integration of a meticulous assessment encompassing both clinical and histological features, coupled with FISH analysis targeting *MAML2* rearrangement, emerges as a reliable methodology. This comprehensive approach is instrumental in distinguishing WL-MEC from various benign and malignant lesions, with particular emphasis on its differentiation from metaplastic WT. The adoption of such diagnostic strategies is imperative for accurate classification and subsequent therapeutic decision-making in the clinical management of this distinctive pathological entity.

## Data availability statement

The original contributions presented in the study are included in the article/supplementary material. Further inquiries can be directed to the corresponding author/s.

## Ethics statement

The studies involving humans were approved by Ethical Review Board of the First Affiliated Hospital of Kunming Medical University. The studies were conducted in accordance with the local legislation and institutional requirements. The participants provided their written informed consent to participate in this study. Written informed consent was obtained from the individual(s) for the publication of any potentially identifiable images or data included in this article.

## Author contributions

YY: Conceptualization, Data curation, Investigation, Project administration, Resources, Software, Visualization, Writing – original draft, Writing – review & editing. ZL: Data curation, Conceptualization, Writing – review & editing. YL: Data curation, Writing – review & editing. LW: Data curation, Investigation, Writing – review & editing. JH: Investigation, Writing – review & editing. SL: Investigation, Writing – review & editing. ZH: Supervision, Writing – review & editing. GP: Supervision, Writing – review & editing.
